# Effects of Vitamin D Supplementation on Recurrence of Nasal Polyposis after Endoscopic Sinus Surgery 

**DOI:** 10.22038/ijorl.2019.37766.2241

**Published:** 2020-01

**Authors:** Farnaz Hashemian, Sonya Sadegh, Javaneh Jahanshahi, Mohammad Ali Seif Rabiei, Farshad Hashemian

**Affiliations:** 1 *Department of Otolaryngology-Head and Neck Surgery, School of Medicine, Hamadan University of Medical Sciences, Hamadan, Iran.*; 2 *Department of Health Sciences, School of Public Health, Hamadan University of Medical Sciences, Hamadan, Iran.*; 3 *Department of Clinical Pharmacy, Faculty of Pharmacy, Tehran Medical Sciences, Islamic Azad University, Tehran, Iran.*

**Keywords:** Chronic rhinosinusitis, Endoscopy, Nasal polyposis, Vitamin D3

## Abstract

**Introduction:**

Chronic rhinosinusitis with nasal polyposis (CRSwNP) is a relatively common disease with serious impacts on patient quality of life. Recurrence of polyps after functional endoscopic sinus surgery (FESS) is a dilemma. Vitamin D3 (VD3) is known to inhibit the proliferation of nasal polyp-derived fibroblasts. The present study aimed to investigate the effects of oral VD3 on the recurrence of polyposis after FESS.

**Materials and Methods:**

This triple-blind placebo-controlled clinical trial was conducted on 40 patients with CRSwNP who did not respond to medical treatment and were candidates for FESS. In addition, the patients had VD3 insufficiency. Following the surgery, all the patients received routine treatment (i.e., fluticasone spray, irrigation, cefixime 400 mg daily for 10 days, and montelukast for a month). Moreover, the case group received oral VD3 tablets 4000 IU (single daily dose) for a month, and the control group received placebo in the same manner. The Sino-Nasal Outcome Test (SNOT-22) and Meltzer endoscopic grading scores were recorded at months 1, 3, and 6 after the study.

**Results:**

In this study, 6 months following the intervention, the severity of polyposis was reported to be significantly lower in the VD3 group compared to the placebo group based on SNOT-22 (16.25±10.16 in the VD3 group vs. 47.45±13.55 in the placebo group; P<0.001) and Meltzer scores (0.50±0.60 in the VD3 group vs. 2.65±0.93 in the placebo group; P<0.001). No adverse effects were observed in the case group.

**Conclusion:**

This study showed the efficacy and safety of vitamin D supplementation in the reduction of polyposis recurrence after FESS in patients with CRSwNP.

## Introduction

Chronic rhinosinusitis (CRS) is a common condition distinguished by mucosal inflammation in the nose and sinuses that continues for more than 3 months and is subdivided to chronic rhinosinusitis with nasal polyposis (CRSwNP) and chronic rhinosinusitis without nasal polyposis (CRSsNP) (1). In the treatment of CRS, functional sinus surgery is generally reserved for those patients whose symptoms persist following appropriate medical therapy. As many patients undergo surgical therapy each year, the treatment of CRS has immense public health implications (2). Various interactions between environmental factors and host immune system are known to play an integral role in the etiology of CRS. However, there is still controversy regarding the etiology and pathophysiology of CRS (3).

CRSsNP is distinguished by elevated T-helper 1 and T-helper 2 (Th2) cytokines with the infiltration of neutrophils and eosinophils. Indeed, CRSwNP exhibits a Th2-skewed immune profile with increased mast cells and eosinophils (4,5).

Increased eosinophils are among the most characteristic features of CRSwNP, and the level of mucosal eosinophilia has a correlation with more substantial sinonasal diseases and lowered likelihood of surgical success (6,7).

Immunoreactivity of Regulated Upon Activation Normal T Cell Express Sequence (RANTES) and release of eosinophil cationic protein are identified in nasal polyp specimens, and they are localized predominantly to the epithelial or endothelial cells (8-11). Another role of RANTES is the mobilization of eosinophils into nasal polyp tissues (10).

Topical steroids are most commonly used for CRSwNP; however, the result is occasionally unsatisfactory, and frequent recurrences occur, and surgical interventions are often mandatory (12). Vitamin D3 (VD3) is a secosteroid hormone that regulates calcium and bone homeostasis and has immunomodulatory effects on monocyte-macrophages and T cells (13). VD3 has also antiproliferative and antiinflammatory effects and plays a crucial role in respiratory health (14,15). In addition, vitamin D derivatives inhibit the proliferation of nasal polyp-derived fibroblasts (8,11). On the other hand, VD3 deficiency is inversely correlated with the infections of the upper respiratory tract, and the higher degrees of VD3 are associated with decreased possibility of asthma-related complications, as well as reduced use of antiinflammatory medication (16). The present study aimed to assess the possible efficacy of Vitamin D supplementation (4000 IU/day) on the recurrence rate of nasal polyposis following functional endoscopic sinus surgery (FESS) in CRSwNP patients. To the best of our knowledge, to date, no one has investigated the efficacy of VD3 in the reduction of the recurrence rate of nasal polyposis following FESS in a triple-blind placebo-controlled trial.

## Materials and Methods

This randomized triple-blinded controlled trial was conducted at Besat Hospital, affiliated with Hamadan University of Medical Sciences, Hamedan, Iran, from September 2016 to November 2017. The study protocol was approved by the Ethics Committee of Hamadan University of Medical Sciences (IR.UMSHA.REC.1395.290) and performed in accordance with the Helsinki Declaration of 1975, revised in 2000. Informed written consent was obtained from all the patients prior to the study. Moreover, the present study was registered at the Iranian Registry of Clinical Trials (IRCT201608043186N8).

Patients suffering from CRS who referred to Besat hospital were entered the study if they met the following inclusion criteria: 

1) Being older than 18 years.

2) Having diagnostic criteria for CRSwNP based on the American Academy of Otolaryngology-Head and Neck Surgery criteria.

3) Having failed standard medical treatment and being indicated for bilateral FESS.

4) Having 10-30 ng/ml serum levels of VD3 (i.e., VD3 Insufficiency). In addition, the exclusion criteria were as follows: 

1) Being sensitive to vitamin D3.

2) Being smokers.

3) Being pregnant.

4) Having long-term systemic steroids use (i.e., more than 10 days).

5) Using medications, such as estrogen, thiazide diuretics, digoxin, antacids, isoniazid, and immunosuppressive drugs.

6) Having a history of any known systemic disorders, such as endocrine, skeletal, kidney and gastrointestinal diseases. Moreover, the sample size was determined according to previous studies (17,18). On the basis of sample size estimation formula in two independent study groups regarding alpha and beta as 0.05 and 0.2, respectively, and an effect size of 0.35 (with the expectation of differences in polyposis recurrence between the two groups), the present study obtained a sample size of 20 for each group and a total sample size of 40 to achieve a significant result with a power of 80% (P<0.05). 

The eligible patients were randomly assigned into two groups using block randomization method (i.e., 4 participants in each block). Block randomization method works by randomizing the patients within blocks in a way that an equal number is assigned to each group. Allocation continued by randomly choosing one of the orderings and assigning the next block of patients to the study groups. In this way, study groups were equal in size and uniformly distributed by principal outcome-related characteristics. Furthermore, patients with aspirin-exacerbated respiratory disease (AERD) and those undergoing revision surgery were randomly distributed in both groups. 

Before the surgery, all the patients received 10 mg of oral prednisolone for 10 days and 400 mg of cefixime per day with two puffs of fluticasone spray in each nostril once a day. The patients underwent endoscopic sinus surgery by two senior surgeons. After the surgery, both groups of patients received routine postoperative management (i.e., debridement in the first week, two puffs of fluticasone spray daily, irrigation, cefixime 400 mg daily for 10 days, and montelukast) for a month. The intervention group received oral vitamin D tablets 4000 IU (single dose daily) for a month (18), and the control group was given placebo in the same manner. The Sino-Nasal Outcome Test (SNOT-22) and Meltzer endoscopic grading scores were recorded before the intervention and at the end of the first, third, and sixth months. Nasal endoscopic findings were categorized according to Meltzer scores as follows: 

0 for no polyps, 1 for small polyps in the middle meatus/edema, 2 for blocked middle meatus, 3 for polyps extending beyond the middle meatus without complete obstruction, and 4 for massive nasal polyposis. The present study was carried out using a triple-blinded design. To this end, a third person (i.e., pharmacologist) provided the identical containers of vitamin D3 and placebo tablets with the same shape and color and coded them. Therefore, neither the patients nor the surgeons were aware of the administered medication. Moreover, the statistical analyst had no knowledge of the study groups before the analysis of the data and decoding of the labels. 

Statistical analysis

Data analysis was performed using SPSS software (version 16.0). Distribution of variables in the two groups was tested for normality by the Kolmogorov-Smirnov test. Quantitative measurements of the two groups were performed using independent sample t-tests (in case of normal distribution). In addition, qualitative comparisons were conducted using Chi-square tests. In all comparisons, p-value less than 0.05 was considered statistically significant.

## Results

**Fig 1:**
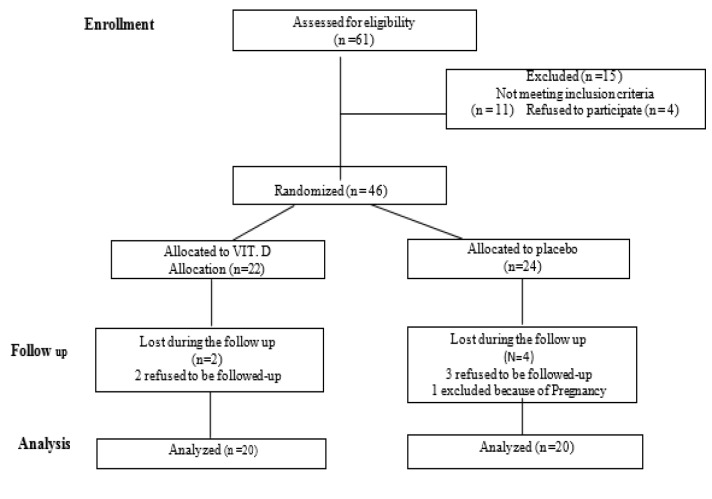
Flowchart of Enrollment and Follow-up Procedure of the Study

Out of 61 patients who initially enrolled, 15 patients were not entered the study (11 patients had no inclusion criteria and 4 patients declined to participate). Therefore, the present study was conducted on a total of 46 patients. Out of 46 cases, 22 and 24 patients received vitamin D (4000 IU) and placebo, respectively. In the drug group, two patients were withdrawn during the follow-ups (voluntarily refused to continue the study). Moreover, in the placebo group, four patients were excluded during the follow-ups (one case due to pregnancy and three patients voluntarily refused to continue the study). Finally, the analysis was performed based on the data obtained from the remaining 40 participants, including 20 in the VD3 group and 20 in the control group. Figure 1 summarizes enrollment and follow-up procedure of the study. The baseline characteristics of the patients are shown in table 1. Mean age of the patients in the placebo and VD3 groups were reported as 42.5±13.79 and 41.35±13.58 years, respectively. Furthermore, six patients with AERD and four patients with revision surgery were randomly assigned to the groups. Prior to the study, the average levels of vitamin D in the placebo and VD3 groups were 17.81 (SD: 4.46) and 17.61 (SD: 4.49), respectively. Therefore, there were no statistically significant differences in VD3 serum levels between the groups prior to the study (P=0.891). Moreover, no statistically significant differences were observed in the severity of sinonasal polyposis between the two groups based on SNOT-22 (P=0.53) and Meltzer (P=0.165) scorings before the intervention (Table.1).

**Table 1:** Participants’ Baseline Characteristics

		**Placebo group**		**VD3 group**		**p-value**
		** Frequency**	**Percent**	**Frequency**	**Percent**	
Gender*	Male	12	60.0	16	80.0	0.168
	Female	8	40.0	4	20.0	
AERD	Yes	3	15.0	3	15.0	1.000
	No	17	85.0	17	85.0	
Revision Surgery	Yes	2	10.0	2	10.0	1.000
	No	18	90.0	18	90.0	
Age (year)**		Placebo group		VD3 group		P-value (diff CI)
		mean	SD	mean	SD	
		42.05	13.79	41.35	13.58	0.87 (-8.06-9.46)
VIT.D level in blood***		17.81	4.64	17.61	4.49	0.89 (-2.67-3.06)
Pre op SNOT-22 score#		67.05	17.44	63.4	19.45	0.53(-8.17-15.47)
Pre op Meltzer score##		3.75	0.55	3.40	0.75	0.16 (-0.07-0.77)

*Chi2: 1.90, df:1, **t: 0.162, df:38, ***t:0.138, df:38,# t:0.625, df:38,##t: 1.67, df:38 AERD: aspirin-exacerbated respiratory disease

After the intervention, endoscopic grading scores were evaluated at three time points, namely the first, third, and sixth months following the study. During the first month, no statistically significant differences were reported between the two groups (P=0.108). However, at months 3 and 6, the VD3 group had significantly lower endoscopic scores (Table.2; P<0.001). Moreover, there were statistically significant differences between the placebo and VD3 groups regarding the severity of symptoms based on the SNOT-22 scoring at months 3 and 6 after the surgery (Table.3; P<0.001). No adverse effects were reported in the study groups.

**Table 2:** Endoscopic Meltzer Scoring in Control and Intervention Groups

	Placebo group		Vit.D tablet group		p-value (diff CI)
	MEAN	SD	MEAN	SD	
Pre-OP Meltzer score*	3.75	0.55	3.40	0.75	0.16 (-0.07-0.77)
Post-OP Meltzer score month 1**	0.30	0.47	0.00	0.00	0.11 (0.08-0.51)
Post-OP Meltzer score month 3#	1.35	0.93	0.20	0.41	< 0.001 (0.68-1.61)
Post-OP Meltzer score month 6##	2.65	0.93	0.50	0.60	< 0.001(1.64-2.65)

*t: 1.67, df:38, **t:2.85, df:38, #t: 5.04, df:38, ##t:8.63, df:38

**Table 3:** SNOT-22 Scoring in Drug and Placebo Group

	Placebo group		Vit.D tablet group		p-value (diff CI)
	MEAN	SD	MEAN	SD	
Pre-OP SNOT-22 score*	67.05	17.44	63.4	19.45	0.53(-8.17-15.47)
Post-OP SNOT-22 score month 1**	18.80	14.05	12.55	9.57	0.10 (-1.44-13.94)
Post-Op SNOT-22 score month 3#	34.70	14.29	14.35	8.64	< 0.001(12.78-27.91)
Post-Op SNOT-22 score month 6##	47.45	13.55	16.25	10.16	< 0.001(23.53-38.86)

t:0.925, df:38, **t:1.64, df:38, #t:5.44, df:38, ##:t:8.63, df:38*

## Discussion

Nasal polyps are the most common benign nasal masses from which the patients have various complaints, such as unilateral or bilateral obstruction, sleep disorders, and poor quality of life (19).

Endoscopic sinus surgery is reported to be the most effective treatment of rhinosinusal polyposis. However, the removal of the entire microscopic inflammatory tissues is not possible, and the residue of nasal and sinusal inflammatory tissues may lead to the recurrence of polyps (2). Therefore, multiple nonsurgical therapies, such as systemic and topical steroids, have been suggested to reduce the postsurgical recurrence of polyposis (20). Recently, it has been shown that VD3 deficiency is correlated with the presence of nasal polyps in CRS patients (21,22). As a result, VD3 supplementation which has antiproliferative and antiinflammatory properties is suggested to be used as an adjunct therapy to decrease the incidence of inflammation and polyposis. To the best of our knowledge, to date, no one has investigated the efficacy of VD3 in the reduction of the recurrence rate of nasal polyposis following endoscopic sinus surgery in a triple-blind placebo-controlled trial. Results of the present study showed the efficacy and safety of vitamin D supplementation in reducing the recurrence of polyposis after endoscopic sinus surgery in patients with CRSwNP. Findings of the present study are in line with the results of several studies exploring possible effects of VD3 supplementation on CRSwNP. Several studies attempted to investigate possible relationships between the serum levels of VD3 and symptoms of patients with AFRS and CRSsNP. For instance, Mostafa et al. compared VD3 serum levels in 74 patients with AFRS, CRS, and CRSwNP. In the aforementioned study, it was shown that VD3 levels in patients with CRSwNP and AFRS were significantly lower than those in patients with CRSsNP and control group. Therefore, in line with the results of the present study, they concluded that the use of VD3 supplementation may be an inexpensive and cost-effective prophylactic measure in the therapeutic control of AFRS and CRSwNP either by itself alone or as a synergistic therapeutic option with traditional therapies (23). In another study, Carrol and Schlosser evaluated the blood and tissue of sinuses from 15 patients with CRSwNP and demonstrated that vitamin D deficiency is accompanied by the elevated proliferation of human sinus fibroblasts in CRSwNP. In their study, the control group consisted of 12 patients who underwent surgery to repair cerebrospinal fluid leakage or secretory hypophysis tumors. When treated with calcitriol tablets, a significant decrease was reported in the fibroblast proliferation index in patients with CRSwNP (P<0.01); however, it was not observed in the control group (24).

In addition, Sansoni *et al. *showed a significant negative correlation between VD3 and basic fibroblast growth factor in CRSwNP patients. Similar to the findings of the present study, they concluded that VD3 is a regulator of fibroblast growth factor in CRSwNP (25). In a retrospective study, Schlosser *et al.* investigated the effect of vitamin D deficiency on clinical presentations in patients with CRSwNP. According to the results of the aforementioned study, 55% of the patients had insufficient VD3 levels. Furthermore, the low levels of VD3 were associated with more severe mucosal disease in computed-tomography scans, which is in line with the findings of the present study (26). Faruk *et al.* studied the efficacy of low-dose (1000 IU/day) and high-dose (4000 IU/day) VD3 in relieving the disease symptoms and reducing the size of nasal polyps. In the aforementioned study, almost similar to the results of the present study, the group that received high-dose VD showed significant decrease in all the symptoms of visual analogue scale (VAS) and endoscopic scores. Therefore, it was concluded that high-dose VD3 supplementation is probably effective in relieving the symptoms of nasal polyposis and restoring the nasal mucosa after the surgery (18). In the present study, subjective symptoms and endoscopic scores decreased in both study groups; however, the levels of decrease in the mentioned scores were reported to be significantly higher in the vitamin D group in comparison to those in the placebo group. Moreover, a systematic review was carried out with the aim of investigating possible relationships between serum levels of VD3 and CRS phenotype and disease severity. A statistically significant relationship was observed between serum VD3 levels and polypoid CRS phenotypes that is consistent with the findings of the present study. Nevertheless, it was suggested to perform further studies on the possible relationships between the levels of VD3 and severity of the disease and VD3 potential for pharmacotherapy (27).

In this randomized triple-blinded placebo-controlled clinical trial, 20 patients received oral vitamin D tablets, 4000 IU (single dose daily) for a month. In addition, 20 patients in the control group were given placebo in the same manner. It is noteworthy that the cases with VD3 insufficiency were included in the present study due to the consideration of the Ethics Committee. Severity of polyposis was significantly lower in the VD3 group, compared to that in the placebo group according to SNOT-22 scoring at all follow-up time points, except for the first month. 

Moreover, Meltzer endoscopic grading scores were significantly lower in the VD3 group in comparison to those in the placebo group at months 3 and 6 after the operation but not significantly different in the first month. This finding may be due to the correction effect of VD3 levels and consequent regulation of fibroblast growth factor. However, in the group receiving VD3, the lower SNOT-22 scores at the third and sixth months, as well as endoscopic grading scores at all follow-ups, were associated with a lower level of nasal polyps recurrence.

## Conclusion

Results of the present study showed the efficacy and safety of vitamin D supplementation in reducing the recurrence of polyposis following endoscopic sinus surgery in patients with CRSwNP. This study was unique in the fact that most probably no one has investigated the efficacy of VD3 in the reduction of recurrence rate of nasal polyposis following endoscopic sinus surgery in a triple-blind placebo-controlled trial. Furthermore, the design of the present study allowed the patients not to be deprived of their routine medical therapies. Nevertheless, further clinical trials are required to be conducted in order to explore whether modifications in medication dose and course of treatment could ameliorate the clinical symptoms and objective outcomes of the patients with CRSwNP following endoscopic sinus surgery.
